# Screening of Estrogenic-Disrupting Compounds in Dairy Products Based on the Estrogen Receptor Cocktail

**DOI:** 10.3390/foods11091178

**Published:** 2022-04-19

**Authors:** Xiaoqi Li, Shuyuan Du, Fangyuan Tian, Minglu Wang, Ying Wang, Hongyan Zhang, Liguo Zang

**Affiliations:** 1 Shandong Provincial Key Laboratory of Animal Resistance Biology, Key Laboratory of Food Nutrition and Safety of Shandong Normal University, College of Life Science, Shandong Normal University, Jinan 250014, China; lixiaoqi_7@163.com (X.L.); dusy@sdnu.edu.cn (S.D.); fanguyuan@163.com (F.T.); w_ml2020@163.com (M.W.); zhanghongyan@sdnu.edu.cn (H.Z.); 2 College of Food Science and Engineering, Shandong Agricultural University, Tai’an 271018, China; wing_0125@163.com

**Keywords:** estrogenic-disrupting compounds, estrogen receptor cocktail, solid phase extraction, gold nanoparticles colorimetric assay, dairy products

## Abstract

The residue of estrogenic-disrupting compounds (EDCs) that are secreted by cows, added as drugs, and present in the feed may exist in dairy products. A gold nanoparticles (AuNPs)-estrogen receptor (ER) cocktail colorimetric assay equipped with ER cocktail solid phase extraction (SPE) was established to screen EDCs. Nine EDCs with high, moderate, and low estrogenic activity were selected to be the representative targets. The recognition range of the colorimetric assay combined with the ER cocktail SPE was wider than that of a single ERα or ERβ. The lowest detection limit of the established assay was about 10^−9^ mg·mL^−1^. The detection limits of estrone, bisphenol A, and bisphenol B were about one order of magnitude lower than the method based on a single ER. The recoveries of the spiked nine EDCs were between 80.0% and 110.0%, and daidzein was identified in the dairy product. The developed method has potential application prospects in food safety and environmental monitoring.

## 1. Introduction

Estrogenic-disrupting compounds (EDCs), a class of exogenous substances with diverse nature and persistence, have the simulated function of endogenous hormones [1], which disrupt the metabolism and reproductive system of animals and humans even at very low exposure dosage. Various natural estrogens produced by living organisms and synthetic numerous chemical compounds used in medicine, industry, and agriculture have been identified as the main sources of EDCs [2]. EDCs have been verified in milk [3], including endogenous natural estrogens secreted by dairy cows (e.g., estradiol, estrone (E1)), hormones as drugs, phytoestrogens (e.g., genistein (GS), daidzein), and mycotoxins with estrogenic activity (e.g., zearalenone) present in the feed. The residue of these estrogenic active compounds in milk/dairy products can disturb the normal physiological function of the human body and even induce cancers [4,5]. Therefore, screening and identification of EDCs in dairy products is of great significance in the field of food safety and environmental monitoring.

The biologically-based assay developed based on whole organisms, whole cells, or biological materials, was the main method for EDC screening. Although the true impact of EDCs on the target species in real environments can be assessed, the whole organism assay lacks specificity in response to different EDCs. The yeast two-hybrid system and proliferation assay using breast cancer cell lines, etc., is a classical biological screening method of EDCs in food and environmental samples. However, the susceptibility of cells to EDC toxicity is an urgent problem to be solved in screening. Compared with the above complex screening methods, many rapid and simple cell-free methods have been applied in the screening of EDCs, such as enzyme-linked receptor assay (ELRA), enzyme-linked immunosorbent assay (ELISA), and gold nanoparticle (AuNPs) colorimetric assay [6,7]. The antibody, aptamer, and single estrogen receptor (ER) α were usually used in molecular recognition to recognize EDCs in rapid screening methods. However, antibodies and aptamers could only recognize the specific target, which limits their application in screening unknown EDCs.

The ER, a kind of nuclear receptor belonging to the steroidal hormone receptor family, includes ERα and Erβ, with the advantage of non-targeted biorecognition [8]. Various methods based on ERα have been established for unknown EDC screening, such as the competitive binding assay [9] and direct binding assay system [10], but the recognition range of a single ER (mainly ERα) was not wide enough. Brennan, J. C. et al. [11] established a recombinant human ovarian cell line with an ERα and ERβ expression plasmid. The recombinant cell line induced luciferase activity by EDCs and confirmed that the dual-ER (ERα and ERβ) cell bioassay can achieve the comprehensive screening of EDCs. About 59% of the amino acid sequence of ERα and ERβ, two subtypes of ER [12], is consistent in the COOH terminal ligand binding domain. However, there are slight differences in the ligand binding sites of alleged fathers-2 on ERα and ERβ [13], so the selectivity of ligands is different. Therefore, the development of a rapid and simple screening method based on ER cocktails composed of ERα and ERβ is expected to improve the screening performance.

The trace EDCs in dairy products are very difficult to extract and analyze due to the complex matrix, which is the main challenge to improving the screening performance. Suitable trace EDC extraction steps and purification techniques are important to remove potentially interfering matrix. The conventional sample preparation usually involves complex sample pretreatment steps, mainly including protein precipitation, fat removal, potential EDC enrichment, and purification. Solid phase extraction (SPE) was usually chosen to enrich trace EDCs from the complex matrix. Zhang et al. [13] prepared an SPE column using ERα to identify and enrich unknown EDCs as a pretreatment method, which was applied successfully in combination with high-performance liquid chromatography (HPLC). However, enlargement of the SPE column enrichment range to recognize more EDCs is of high demand and challenging. This study used the ER cocktail as recognition molecules to achieve a wider recognition range and enrich more EDCs in the sample pretreatment step.

**Scheme 1:**
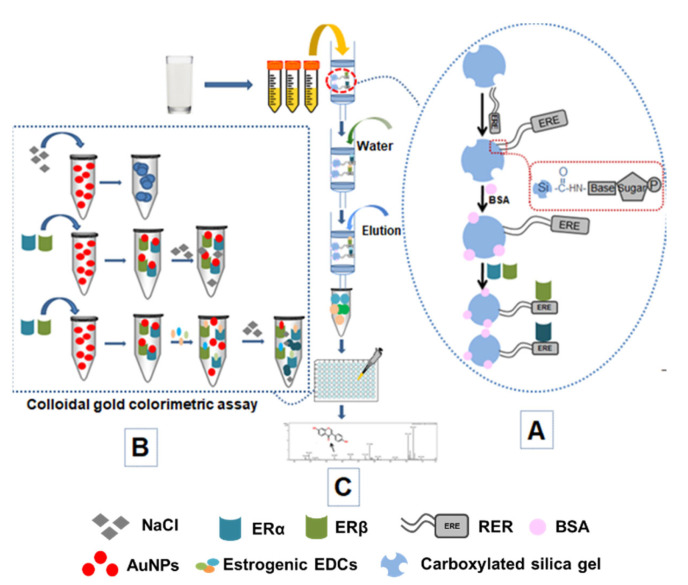
Schematic diagram of AuNPs-ER cocktail colorimetric assay equipped with SPE based on ER cocktail. (**A**) Preparation of the element-based SPE column based on ER cocktail. (**B**) Schematic illustration of the developed AuNPs-ER cocktail colorimetric assay. (**C**) The trace EDCs in dairy products were enriched and purified using ER cocktail SPE column.

A AuNPs-ER cocktail colorimetric assay equipped with an SPE column based on an ER cocktail was designed to rapidly screen EDCs in the complex food matrix. The ER cocktail can protect AuNPs from a salt-induced aggregation [8,14] in a colorimetric assay that uses AuNPs as colorimetric probes. An SPE column was prepared to enrich and purify EDCs in the sample based on the immobilized ER cocktail by the estrogen response element (ERE) that maintains maximum ER activity (Scheme 1A). For the positive samples, the ER cocktail can bind specific purified EDCs, which makes AuNPs lose the protection of the ER cocktail and a large quantity of salt approach the AuNPs to induce their aggregation (Scheme 1B). The characteristic absorption peak of AuNPs was shifted as the aggregation took place, and then the absorbance values ratio of before and after aggregation was calculated to detect EDCs. Moreover, a substance with estrogenic activities was screened, separated, and identified by mass spectrometry (Scheme 1C). Accordingly, the prepared SPE column using the ER cocktail could enrich trace amounts of EDCs and reduce the interference of matrix in dairy products. The developed AuNPs-ER cocktail colorimetric assay realized high-throughput analysis of known EDCs and screening of unknown EDCs in dairy products. Compared with the recombinant cell lines with the dual-ER (ERα and ERβ) method established by Brennan, J. C. et al. [11] for EDC screening, this proposed method was more convenient, time-saving, and achieved a wider recognition range for EDC screening.

## 2. Materials and Methods

### 2.1. Reagents and Instruments

Human ERα and ERβ were purchased from Wuhan Pujian Biotechnology Co., Ltd. (Wuhan, China). Diethylstilbestrol (DES), 17α-estradiol (17α-E2), GS, hexestrol (HES) were brought from Macklin Biochemical Co., Ltd. (Shanghai, China). Daidzein and 17β-estradiol (17β-E2) were obtained from Ryon Biologial Technology Co., Ltd. (Shanghai, China). Bisphenol B (BPB) and E1 were purchased from Yuanye Biologial Technology Co., Ltd. (Shanghai, China). 2,2-bis (4-hydroxyphenyl)-1,1,1-trichloroethane (HPTE) and bisphenol A (BPA) were brought from Aladdin Biochemical Technology Co., Ltd. (Shanghai, China). Trisodium citrate dihydrate was purchased from Tianjin Huate Chemical Research Technology Co., Ltd. (Tianjin, China). Chloroauric acid (HAuCl_4_), sodium chloride (NaCl), and methanol were brought from Sinopharm Chemical Reagent Co., Ltd. (Shanghai, China). Bovine serum albumin (BSA) was purchased from Solarbio Science & Technology Co., Ltd. (Beijing, China). ERE was prepared by Tsingke Biology Co., Ltd. (Qingdao, China). Triphenylamine and sulfonyl chloride were from Maclean Biochemical Technology Co., Ltd. (Shanghai, China). Rotary evaporator (N-1100, RIKAKIKAI, Tokyo, Japan), centrifuge tubes, 96-well microplates were from Jinan Chengsen Trading Co., Ltd. (Jinan, China). Ultrapure water, carboxylated silica gel, and empty SPE columns were made in the laboratory. The electric heating plate (HP550-S, Beijing, China), transmission electron microscope (TEM, JEM-100CX II, Tokyo, Japan), microplate reader (Multiskan Sky, ThermoWaltham, Waltham, MA, USA), Hypersil BDS C18 column, (Waltham, MA, America), Hypersil BDS C18 column (150 × 4.6 mm, 5 μm, Thermo Fisher Scientific, Shanghai, China), water purification system (Milli-Q, Bedford, MA, USA), and HPLC (U3000, Thermo Fisher Scientific, Shanghai, China) were used and analyzed in the laboratory.

### 2.2. Preparation of SPE Column

SPE columns were prepared using ERα, ERβ, and ER cocktail as recognition molecules according to reference with minor modification [13]. The silica gel was acidified using HCl, chlorinated with sulfonyl chloride dissolved in anhydrous pyridine, and mixed with adipic acid, toluene, and pyridine to produce the carboxylated silica gel. Ten milligrams of activated carboxylated silica gel was combined with 66 μL (1 mg·mL^−1^) ERE at room temperature for 2 h. After removing the unbound ERE by washing with ultrapure water that had an electrical conductivity of 18.2 MΩ, 300 µL of BSA (*w*/*v*, 3 %) was added and incubated for 1.5 h at room temperature to block the residual binding sites on the silica gel. After washing away the unbound BSA with ultrapure water, 0.5 mg·mL^−1^ of ERα was incubated with silica gel for 2 h. Then, 300 µL of 0.01 mg·mL^−1^ triphenylamine was used to block the binding sites of small molecules at room temperature, and the silica gel coupled with ERα was transferred to a 2 mL empty SPE column. The SPE columns based on ERβ or ER cocktail were prepared referring to the method described above.

### 2.3. Enrichment of EDCs with SPE Column

One milliliter different concentrations of nine EDCs (17β-E2, 17α-E2, E1, DES, HES, HPTE, GS, BPB, BPA) were slowly injected into the SPE columns using the constant flow pump and incubated with ER immobilized on the SPE columns for 2 h at room temperature. After the unbound EDCs were washed off with ultrapure water, the combined EDCs by ER were eluted from the SPE column by a 40% methanol–water solution.

### 2.4. Preparation of AuNPs

The AuNP solution was prepared by reducing trisodium citrate according to the reference procedure with slight modification [8,15]. An amount of 1 mL HAuCl_4_ solution (*w*/*v*, 1%) and 99 mL ultrapure water were mixed and heated to boiling in a clean beaker washed by aqua regia. After boiling for 3 min, 3 mL trisodium citrate dihydrate (*w*/*v*, 1%) was quickly added into a beaker and kept stirring until the color remained unchanged, then continued stirring for another 10 min. The AuNP solution was stirred to room temperature after removing from heat and preserved in a brown bottle at 4 °C before use.

### 2.5. Optimization of the Concentrations of ERα and ERβ

An amount of 20 μL of ERα with concentrations of 0, 2, 4, 6, 8, 10, 11, 13, 15, 20, 25 μg·mL^−1^ were added into 96-well plates in triplicate, respectively. Then, 40 μL of ultrapure water and 100 μL of AuNP solution were mixed with ERα and reacted for 10 min at room temperature, and then 50 μL of 7.6 mg·mL^−1^ NaCl solution was added and incubated. When the color of the system remained unchanged, the absorbance at 520 nm was recorded by the microplate reader to characterize the optimal ERα concentrations for protecting AuNPs from salt-induced aggregation. The optimization of ERβ concentration was the same as that of ERα.

### 2.6. Preparation of ER Cocktail and Optimization of NaCl Concentration

The original concentrations of ERα and ERβ were diluted to 20 μg·mL^−1^. Equal volumes of 20 μg·mL^−1^ ERα and 20 μg·mL^−1^ ERβ were mixed to keep 10 μg·mL^−1^ ERα and 10 μg·mL^−1^ ERβ for further use, which was named as ER cocktail.

When the added NaCl exceed the optimal concentration, a sharp decrease of the absorbance value due to AuNP aggregation was observed, so the NaCl around the optimized concentration was optimized in detail. Then, 20 μL of ER cocktail was diluted 3 times using ultrapure water and added into 96-well plates in triplicate, then 100 μL of AuNP solution was added and incubated for 30 min. An amount of 40 μL of NaCl solutions of different concentrations of 0, 9, 18, 23, 26, 29, 41, and 53 mg·mL^−1^ were added and reacted until the color remain unchanged. The absorbance at 520 nm was recorded by a microplate reader to determine the appropriate concentration of NaCl that makes AuNPs just unaggregated under the protection of ER cocktail.

### 2.7. Determination of EDCs by AuNPs-ERα/ERβ/ER Cocktail Colorimetric Assay

Nine EDCs at different concentrations (10^−9^, 10^−8^, 10^−7^, 10^−6^, 10^−5^, 10^−4^, 10^−3^, 10^−2^ mg·mL^−1^) were reacted with ERα/ERβ/ER cocktail for 2 h at room temperature, then 100 μL of AuNP solution was added and incubated for another 10 min. Then, 40 μL of NaCl solution with 26 mg·mL^−1^ was added and reacted until the color remain unchanged. The absorbance values at 630 nm for aggregation state and 520 nm for dispersion state were recorded by the microplate reader to calculate the ratio value of A630/A520 for establishment of the calibration curve.

### 2.8. Enrichment and Detection of EDCs in Dairy Products

Dairy products were purchased from a local supermarket (Jinan, China). Known concentrations of nine EDCs (10^−3^ mg·mL^−1^) were spiked to dairy products without estrogenic effect to evaluate the accuracy of AuNPs-ER cocktail colorimetric assay combined with the SPE column in the real sample.

An amount of 10 mL of acetate buffer (pH 5) and 100 μL of β-glucuronidase (100 Ku) were added in dairy products and incubated at 37 °C overnight to extract the free and bound EDCs [3]. On the second day, acetonitrile was added to dairy products (*v*/*v*, 1:1) to collect EDCs and precipitate protein. Then, the sample was centrifugated at 6000 g for 10 min after vortex, and the precipitation was discarded. One hundred milliliters of N-hexane was completely mixed with supernatant and statically stratified, then the N-hexane layer dissolved lipid was discarded. The liquid without protein and lipid was evaporated by rotary evaporation (60 °C for 30 min), and ultrapure water was added to dissolve the dried sample. The target dissolved in ultrapure water was filtrated through a 0.22 μm filter membrane and slowly injected into the SPE columns based on ER cocktail to enrichment. The potential EDCs in eluent were detected by AuNPs-ER cocktail colorimetric assay according to the procedure described above.

### 2.9. Isolation and Identification of EDCs

The enriched unknown EDCs by SPE columns based on ER cocktail were separated by thin layer chromatography (TLC). The volume ratio 3:1 of methanol and chloroform was chosen as mobile phase in TLC to separate the samples with estrogenic effect [16]. Samples separated by TLC were scraped from the silicone rubber plate and dissolved in methanol, then screened by AuNPs-ER cocktail colorimetric assay. The sample with estrogenic effect screened using colorimetric assay was analyzed and identified by high-resolution orbitrap mass spectrometry [17].

### 2.10. Data Analysis

Data were presented as mean ± standard error (SE). The statistical analysis of the standard curve of nine EDCs and daidzein was drawn in Origin.

## 3. Results and Discussion

### 3.1. Establishment of SPE Column for Sample Pretreatment

**Figure 1:**
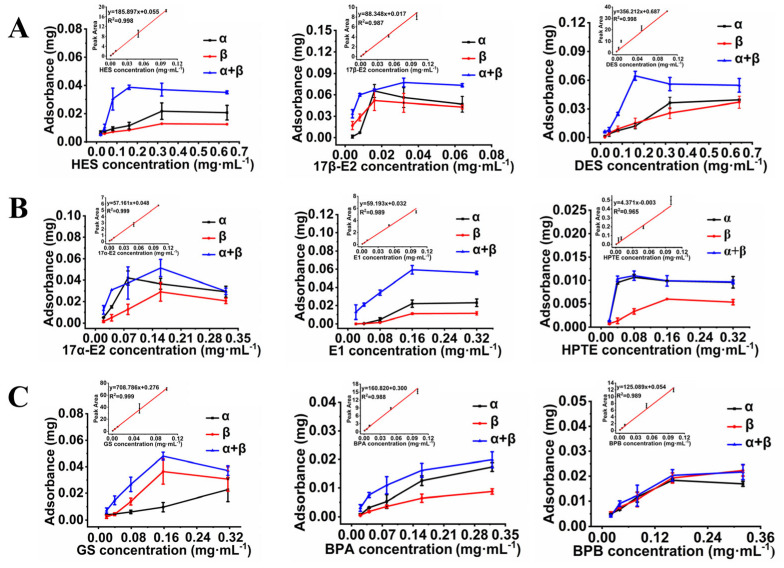
Adsorption capacity of SPE columns prepared with ERα, ERβ, and ER cocktail for EDC adsorption. (**A**) The adsorption capacity of DES, 17β-E2, and HES with high estrogenic activities. (**B**) The adsorption capacity of 17α-E2, E1, and HPTE with moderate estrogenic activities. (**C**) The adsorption capacity of BPA, BPB, and GS with low estrogenic activities. Inset: standard curves of nine EDCs determined by HPLC.

Nine reported EDCs (DES, 17β-E2, HES, 17α-E2, HPTE, E1, BPA, BPB, and GS) with high, moderate, and low estrogenic activities were selected as the representative targets. The standard curves (Figure 1 inset) were obtained by plotting the peak area of HPLC against different concentrations (0.001, 0.005, 0.01, 0.05, 0.1 mg·mL^−1^) of nine EDC standards to calculate the adsorption capacity of SPE columns. SPE columns prepared with ERα, ERβ, and the ER cocktail were used to adsorb nine EDCs, and the adsorption capacity was calculated according to the established standard curve. The adsorption capacity and recognition range of nine EDCs using three types of SPE columns were compared in detail.

In summary, the adsorption capacity and variety of adsorbed EDCs by SPE columns based on the ER cocktail were significantly better than those based on individual ERα or ERβ (Figure 1). For high estrogenic activity EDCs such as DES, 17β-E2, and HES, the adsorption capacities of SPE columns based on ERα were higher than those based on Erβ, but still lower than the adsorption capacities by SPE columns based on the ER cocktail. For moderate estrogenic activity EDCs, the E1 adsorption capacity of SPE columns based on the ER cocktail was significantly higher than that of SPE columns. For low estrogenic activity EDCs, although SPE columns based on the ER cocktail AAshowed the best adsorption effect, GS preferentially bound to Erβ, as shown in Figure 1C, which was consistent with the previous studies [18]. About half of the amino acid sequence of alleged fathers-2 on ERα and ERβ are different. Therefore, there are both common and different ligand binding sites on ERα and ERβ [13], which may be the reason for the synergistic effects of the ER cocktail in ligand recognition. Therefore, SPE columns prepared with the ER cocktail not only have a better adsorption capacity effect on EDCs, but also were conducive to enriching isoflavones in the food or environmental matrix than those prepared with individual ERα or ERβ.

### 3.2. Characterization of Prepared AuNPs

**Figure 2:**
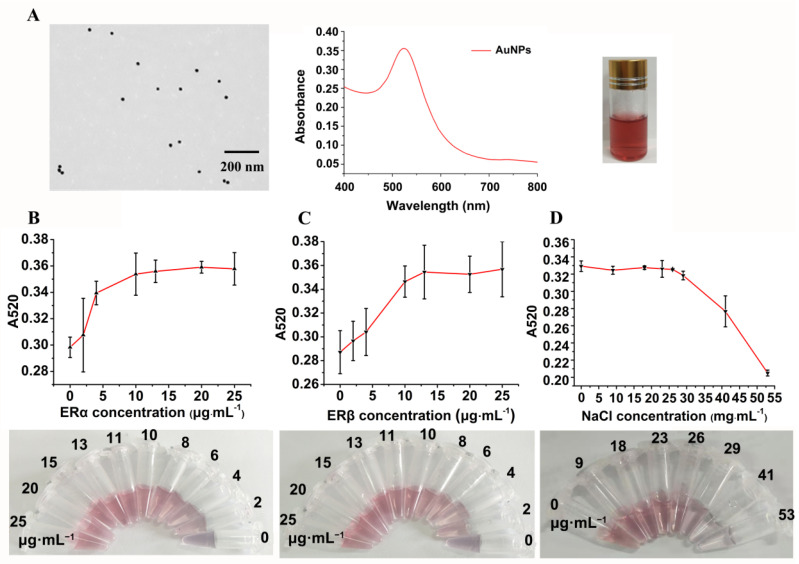
Characterization of AuNPs and optimization of *AuNPs-ER cocktail* colorimetric assay. (**A**) TEM image, UV-visible spectrum, and photograph of the synthesized AuNPs. Optimization of the concentration of ERα (**B**) and ERβ (**C**) by absorbance values at 520 nm and photograph in the presence of 7.6 mg·mL^−1^ NaCl. (**D**) Optimization of the concentration of NaCl added in *AuNPs* in the presence of ER cocktail.

The prepared AuNPs were bright burgundy color, as shown in Figure 2A [19]. The average particle size of the dispersed AuNPs with uniform spherical shape was assessed at about 20 nm through the TEM image. The maximum absorption peak of AuNPs in the UV-visible spectrum was at about 520 nm (Figure 2A), which was usually regarded as the characteristic peak of 20 nm AuNPs [8]. The above results indicated that AuNPs with good dispersion were prepared successfully and could be used in this experiment.

### 3.3. Establishment of AuNPs-ER Cocktail Colorimetric Assay

ER as the protein superfamily attached to AuNPs can protect AuNPs from a salt-induced aggregation. According to the principle of AuNP colorimetric assay [14,20,21], the minimum concentration of ER was optimized to prevent AuNPs from aggregating and improve the sensitivity of detection. Obviously, the A520 of ERα concentration curves in Figure 2B was significantly increased with the increase of ERα concentration and leveled off slowly at the minimum concentration of ERα 10 μg·mL^−1^. On increasing ERα concentration from 0–10 μg·mL^−1^, the color of the AuNPs-ERα solution changed from blue to red, and then stabilized in red as the ERα concentration increased further, which indicated the 10 μg·mL^−1^ of ERα just prevented aggregation of AuNPs at 7.6 mg·mL^−1^ NaCl. Similarly, the A520 gradually increased in the ERβ concentration range of 0–10 μg·mL^−1^, and then did not obviously change, as shown in Figure 2C. As described above, the optimal concentrations of both ERα and ERβ were 10 μg·mL^−1^. Therefore, to ensure the sensitive detection, the optimal concentration of the ER cocktail was also 10 μg·mL^−^^1^, which was prepared by mixing equal volumes of 20 μg·mL^−1^ ERα and 20 μg·mL^−1^ ERβ.

**Figure 3:**
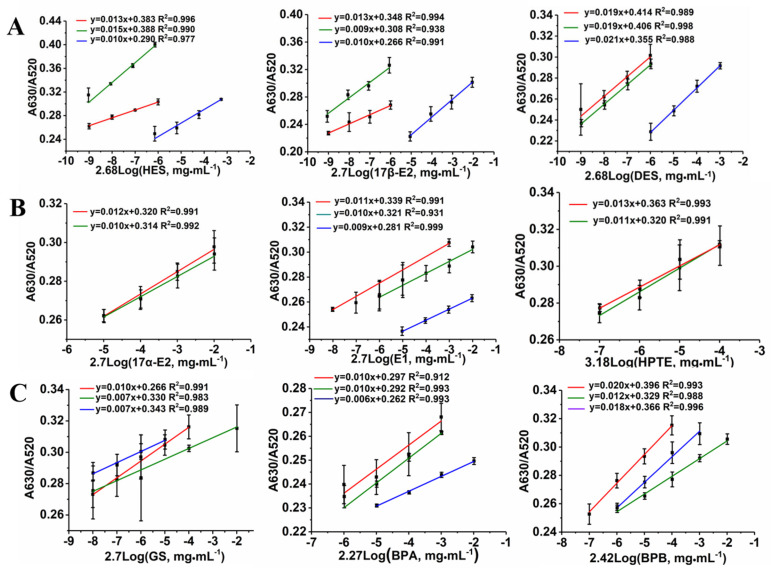
The standard curves of nine different estrogenic activity EDCs including linear range and linear relationship based on different ER colorimetric methods. (“Red line” represents AuNPs-ER cocktail colorimetric assay; “Green line” represents AuNPs-ERα colorimetric assay; “ Blue line ” represents AuNPs-ERβ colorimetric assay). (**A**) The standard curves of DES, 17β-E2, and HES with high estrogenic activities. (**B**) The standard curves of 17α-E2, E1, and HPTE with moderate estrogenic activities. (**C**) The standard curves of BPA, BPB, and GS with low estrogenic activities.

Since the ER cocktail can protect AuNPs from aggregation, the NaCl concentration needs to be optimized so that the color of AuNPs could change sensitively. As illustrated in Figure 3, the absorbance value of AuNPs remained nearly constant with the increased concentration of NaCl in the range of 0–26 mg·mL^−1^, and then a speed decrease was observed, indicating 26 mg·mL^−1^ NaCl just kept the AuNPs from aggregation. Accordingly, induced by high concentrations of NaCl, the AuNPs aggregated into larger sizes and changed to blue color. Therefore, NaCl concentration was optimized at 26 mg·mL^−1^ to make appear the optimum color rendering in the presence of the ER cocktail (Figure 2D).

### 3.4. The Efficiency of AuNPs-ER Cocktail Colorimetric Assay for EDC Detection

Nine EDCs with high, moderate, and low estrogenic activities were tested by AuNP colorimetric assay established based on the different recognition molecules of ERα, ERβ, and ER cocktail. Although the ER protein can prevent nanoparticle aggregation induced by recruiting Na^+^ of NaCl from AuNPs, the adequate EDCs in the sample competed with AuNPs for ER and induced their aggregation. The characteristic absorption peak of AuNPs at 520 nm shifted to 650 nm as the AuNPs aggregated, and the solution color changed from red to blue. Therefore, the absorbance values ratio of A630/A520 was calculated to analyze the number of EDCs by the dispersion and aggregation state of the AuNPs.

The standard curves of nine EDCs were obtained between the A630/A520, as well as different concentrations of EDCs. As illustrated in Figure 3, the detection range of the AuNPs-ER colorimetric assay based on ERβ for BPB and GS detection was better than that based on ERα, which is probably because GS and BPB can preferentially bind to ERβ, consistent with the previous studies. Meanwhile, the detection limit of a colorimetric assay based on the ER cocktail for BPB was an order of magnitude lower than that based on ERα and ERβ (Figure 3C).

In summary, the detection performance of nine different estrogenic activity EDCs including detection limit and detection range of colorimetric assay based on the ER cocktail were significantly better than that of a single ERα or ERβ. The detection category of the AuNPs-ER cocktail colorimetric assay was much richer than that of single ERβ, which can realize the detection of the nine representative EDCs. The detection limits of the AuNPs-ER cocktail colorimetric assay were calculated to be 2.7 × 10^−9^ mg·mL^−1^ for 17β-E2, HES, and DES, 2.7 × 10^−8^ mg·mL^−1^ for E1 and GS, 2.7 × 10^−7^ mg·mL^−1^ for 17α-E2, 3.18 × 10^−7^ mg·mL^−1^ for HPTE, 2.42 × 10^−7^ mg·mL^−1^ for BPB, and 2.27 × 10^−6^ mg·mL^−1^ for BPA (Figure 3C). Therefore, it was confirmed that the detection limit of a colorimetric assay based on the ER cocktail was obviously lower than that based on ERβ, and was a little lower than that based on ERα for a certain category.

### 3.5. Performance Assessment of the AuNPs-ER Cocktail Colorimetric Assay Equipped with an ER Cocktail SPE Column

**Figure 4:**
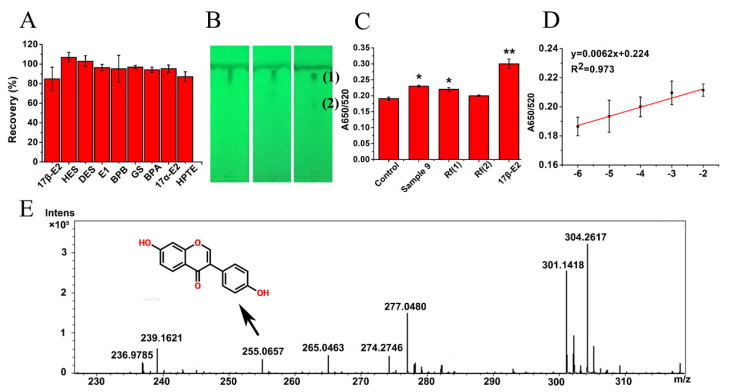
(**A**) The recovery results of milk spiked into nine EDCs analyzed by AuNPs-ER cocktail colorimetric assay. (**B**) The separation of unknown EDCs by TLC from dairy products. (**C**) Estrogenic activity of EDCs in dairy products demonstrated by AuNPs-ER cocktail colorimetric assay. (* indicate statistically significant differences between groups (*p* < 0.05), *n* = 3, ** indicate statistically significant differences between groups (*p* < 0.01), *n* = 3. (**D**) The standard curve of daidzein was obtained by AuNPs-ER cocktail colorimetric assay. (**E**) The mass spectrometry of unknown EDC substances with higher estrogenic activity isolated from dairy products.

To further evaluate the practicality of the designed AuNPs-ER cocktail colorimetric assay, the representative nine EDCs of 10^−3^ mg·mL^−1^ spiked in milk samples were measured. As shown in Figure 4A, the recoveries of the nine spiked milk sample EDCs were all between 80% and 110%. The results clearly demonstrated that the AuNPs-ER cocktail colorimetric assay equipped with an ER cocktail SPE column has good accuracy and can meet the application requirements in real samples of rapid detection technologies (Figure 4A).

Moreover, the SPE column and AuNP colorimetric assay based on the ER cocktail were applied to screen EDCs in different dairy products. The unknown EDCs in the real sample were separated and enriched in a sample pretreatment procedure using the ER cocktail SPE column. The sample of No. 9, soy milk powder, showed an estrogenic effect according to the screening results of the AuNPs-ER cocktail colorimetric assay (Figure 4C). Then, the substances with estrogenic effects in sample No. 9 were separated by TLC using methanol and chloroform (*v*/*v*, 1:3) as spread solvents (Figure 4B). All substances of sample No. 9 separated by TLC were scraped separately from the silicone rubber plate and dissolved in methanol, and then detected by AuNPs-ER cocktail colorimetric assay. It was found that the substance one of sample No. 9 (retention factors: Rf (1) = 0.83) had higher estrogenic activity than substance two (Rf (2) = 0.62) (Figure 4C). Therefore, it was confirmed that the AuNPs-ER cocktail colorimetric assay can be used as a screening method for tracing EDCs in dairy products.

The isolated and purified substance one of sample No. 9 was analyzed by mass spectrometry and was identified at *m*/*z* 255.0654 (Figure 4E). Since sample No. 9 is soy milk powder, substance one might be daidzein, according to the *m*/*z*. Daidzein is one kind of soybean isoflavone (SI) with biological activity and weak estrogenic effects [22]. The standard curves of daidzein were obtained between the A630/A520 detected using AuNPs-ER cocktail colorimetric assay and different concentrations of daidzein. According to the standard curve of daidzein (Figure 4D), the amount of daidzein was calculated at about 17.4 mg per packet of sample No. 9. Studies [23] have shown that isoflavones have no impact on health when adults intake no more than 40 mg/day for a year and no more than 100 mg/day for a month. Furthermore, there is an increased risk of inducing tumors if women intake more than 100 mg/day of isoflavones for one year. Therefore, the amount of daidzein in the daily consumption of sample No. 9 would not have an unacceptable adverse effect on the human body.

## 4. Conclusions

In the study, an AuNPs-ER cocktail colorimetric assay for a screen of unknown EDCs was developed and equipped with ER cocktail SPE. The prepared SPE column used the ER cocktail to separate and enrich as much potential EDCs as possible for improving the detection performance of the colorimetric detection method. The recognition range of this designed colorimetric assay based on the ER cocktail was wider than the assay based on a single ERα or ERβ. Furthermore, the detection limits of the AuNPs-ER cocktail colorimetric assay were from 2.7 × 10^−9^ mg·mL^−1^ to 2.27 × 10^−6^ mg·mL^−1^ for nine representative targets, which showed the detection sensitivity was increased for some categories compared with a colorimetric assay based on a single ER. EDCs in dairy products were isolated and screened using the newly developed method, and daidzein in sample No. 9 was detected and identified. The study provides a method to rapidly screen unknown EDCs in the complex food matrix, such as dairy products, and evaluate the safety of foods containing EDCs.

## Data Availability

Data is contained within the article.

## Abbreviations

**EDCs**	estrogenic-disrupting compounds
**AuNPs**	gold nanoparticles
**ER**	estrogen receptor
**SPE**	solid phase extraction
**E1**	estrone
**GS**	genistein
**ELRA**	enzyme-linked receptor assay
**ELISA**	enzyme-linked immunosorbent assay
**HPLC**	high-performance liquid chromatography
**ERE**	estrogen response element
**DES**	diethylstilbestrol
**17α-E2**	17α-estradiol
**HES**	hexestrol
**17β-E2**	17β-estradiol
**HPTE**	2,2-bis (4-hydroxyphenyl)-1,1,1-trichloroethane
**BPA**	bisphenol A
**BSA**	bovine serum albumin
**TLC**	thin layer chromatography
**SE**	standard error
**SI**	soybean isoflavones
